# Novel mutation in the CHST6 gene causes macular corneal dystrophy in a black South African family

**DOI:** 10.1186/s12881-016-0308-0

**Published:** 2016-07-20

**Authors:** Nadia Carstens, Susan Williams, Saadiah Goolam, Trevor Carmichael, Ming Sin Cheung, Stine Büchmann-Møller, Marc Sultan, Frank Staedtler, Chao Zou, Peter Swart, Dennis S. Rice, Arnaud Lacoste, Kim Paes, Michèle Ramsay

**Affiliations:** Sydney Brenner Institute for Molecular Bioscience, University of the Witwatersrand, 2050 Johannesburg, Gauteng South Africa; Division of Ophthalmology, Department of Neurosciences, University of the Witwatersrand, Johannesburg, South Africa; Biomarker Development, Novartis Institutes for BioMedical Research, Basel, Switzerland; Center for Proteomic Chemistry, Novartis Institutes for BioMedical Research, Basel, Switzerland; Division of Anatomical Pathology, National Health Laboratory Services and University of the Witwatersrand, Johannesburg, South Africa; Novartis Institutes for Biomedical Research, Cambridge, USA; Division of Human Genetics, National Health Laboratory Service and Faculty of Health Sciences, University of the Witwatersrand, 2050 Johannesburg, Gauteng South Africa

**Keywords:** Macular corneal dystrophy, CHST6, Whole exome sequencing, African

## Abstract

**Background:**

Macular corneal dystrophy (MCD) is a rare autosomal recessive disorder that is characterized by progressive corneal opacity that starts in early childhood and ultimately progresses to blindness in early adulthood. The aim of this study was to identify the cause of MCD in a black South African family with two affected sisters.

**Methods:**

A multigenerational South African Sotho-speaking family with type I MCD was studied using whole exome sequencing. Variant filtering to identify the MCD-causal mutation included the disease inheritance pattern, variant minor allele frequency and potential functional impact.

**Results:**

Ophthalmologic evaluation of the cases revealed a typical MCD phenotype and none of the other family members were affected. An average of 127 713 variants per individual was identified following exome sequencing and approximately 1.2 % were not present in any of the investigated public databases. Variant filtering identified a homozygous E71Q mutation in *CHST6*, a known MCD-causing gene encoding corneal N-acetyl glucosamine-6-O-sulfotransferase. This E71Q mutation results in a non-conservative amino acid change in a highly conserved functional domain of the human *CHST6* that is essential for enzyme activity.

**Conclusion:**

We identified a novel E71Q mutation in *CHST6* as the MCD-causal mutation in a black South African family with type I MCD. This is the first description of MCD in a black Sub-Saharan African family and therefore contributes valuable insights into the genetic aetiology of this disease, while improving genetic counselling for this and potentially other MCD families.

**Electronic supplementary material:**

The online version of this article (doi:10.1186/s12881-016-0308-0) contains supplementary material, which is available to authorized users.

## Background

Macular corneal dystrophy (MCD) (OMIM #217800) is a rare autosomal recessive disorder that is characterized clinically by irregularly shaped superficial opacities that progressively extend through the corneal stroma [1, 2]. Onset typically occurs in the first decade of life and progresses to severe bilateral visual impairment in adulthood, which ultimately necessitates keratoplasty [3]. MCD has been identified in a number of populations across the world [4–11], but it has not yet been reported in Sub-Saharan Africa.

Mutations in the carbohydrate sulfotransferase 6 gene (*CHST6*) were identified as the cause for MCD in 2000 [12]. *CHST6* encodes corneal N-acetyl glucosamine-6-O-sulfotransferase (C-GlcNAc6ST), an enzyme which catalyses the sulfation of GlcNAc residues in the main glycosaminoglycan in the cornea, keratan sulfate, to generate sulfated keratan sulfate (KS). The defective sulfation of keratan sulfate that is caused by a deficiency in this enzyme leads to malformations in fibril organization in the cornea, which results in progressive corneal opacification in MCD patients [13–15].

MCD can be divided into two immunophenotypes based on the reactivity of the patient’s serum and corneal tissue to an antibody against KS. Antigenic KS reactivity is very low (or undetectable) in the serum and absent in corneal tissue in MCD type I patients, whereas normal to sub-normal KS levels are detectable in the serum and corneal stroma of type II MCD patients [16]. *In situ* hybridization analysis did not detect *CHST6* transcripts in corneal epithelium of an MCD type II patient, suggesting that the mutations found in type II lead to a loss of cornea-specific expression of *CHST6* [12]. These two subtypes are, however, clinically indistinguishable.

Patients with type I MCD usually harbour missense mutations and indels in the coding region of *CHST6*, which leads to the functional inactivation of the enzyme [12, 17], whereas deletions and/or rearrangements in the region between *CHST6* and the neighbouring *CHST5* have been reported in patients with MCD type II [1, 12]. This region likely harbours a gene regulatory element that affects the cell-specific transcription of *CHST6* [12].

However, some studies failed to find potentially causative mutations in the coding region, upstream regulatory region or splice site mutations of *CHST6* in MCD patients [1, 5, 6, 9, 18, 19]. Current theories to explain this include pathogenic mutations in an unknown regulatory promoter immediately upstream of *CHST6* or cis-acting distant regulatory elements [1, 20], while genetic heterogeneity has not yet been excluded [1].

In the present study, which is the first description of MCD in a sub-Saharan African family, we used whole exome sequencing to identify the MCD-causal mutation in a consanguineous black South African family.

## Methods

### MCD Family

**Fig. 1:**
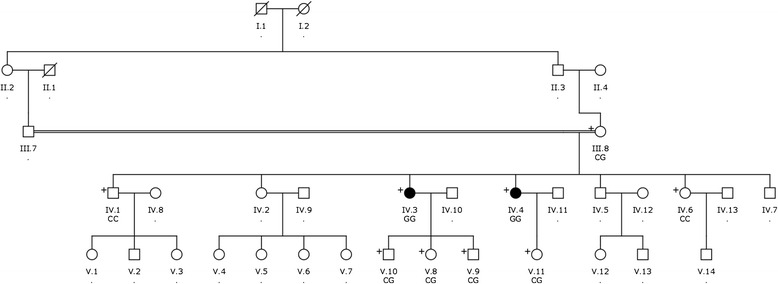
Schematic representation of the MCD pedigree. The 9 sequenced individuals are indicated with a + and their genotype for the E71Q mutation is indicated below the Individual ID. Individuals IV.3 and IV.4 (shaded black) show clinical manifestations of macular corneal dystrophy (MCD), while their consanguineous parents, siblings IV.1 and IV.6 and children did not

Two sisters presented with a complaint of decreased vision in 2007 to the Eye Clinic of the Charlotte Maxeke Johannesburg Academic Hospital in Johannesburg, South Africa. They were diagnosed with MCD based upon the distinctive clinical features, which were later confirmed by histopathologic examination following penetrating keratoplasty. We invited the entire family for an ophthalmic examination and seven additional family members consented to participate in this study. The pedigree was consistent with an autosomal recessive inheritance pattern and showed evidence of consanguinity (Fig. 1).

The study was approved by the Human Research Ethics Committee (Medical) of the University of the Witwatersrand, South Africa (protocol number M131125), and followed the tenets of the Declaration of Helsinki. Written informed consent was obtained from all participating family members for participation in this study and the use of their DNA and clinical data for research purposes after a genetic counsellor explained the nature and possible consequences of the study to them.

Complete ophthalmic examination of each participating family member was performed. Phenotyping included a slit-lamp examination, anterior segment photography and OCULUS Pentacam® examination.

### Whole exome sequencing, assembly and variant calling

Targeted exome capture was performed by preparing sequencing libraries from genomic DNA using the NuGEN Ovation Ultralow DR Multiplex protocol followed by a SureSelectXT Human All Exon V5 + UTRs (70 MB) target enrichment (Agilent, Basel, Switzerland). Captured libraries were sequenced on the Illumina HiSeq 2500 with 76-bp paired-end reads. Reads were mapped to the human hg19 genome assembly using the Burrows-Wheeler Aligner (BWA) [21] and GATK base quality score recalibration, indel realignment, and duplicate removal was applied according to GATK Best Practises guidelines [22]. Variants were called with the GATK Unified Genotyper [23] and annotated with the Ensembl Variant Effect Predictor [24]. Variants with a VQSLOD score below 2 and/or a base coverage below 5x were considered low quality calls and consequently not included in the analysis.

### Variant filtering to identify putative MCD-causal variant(s)

**Fig. 2:**
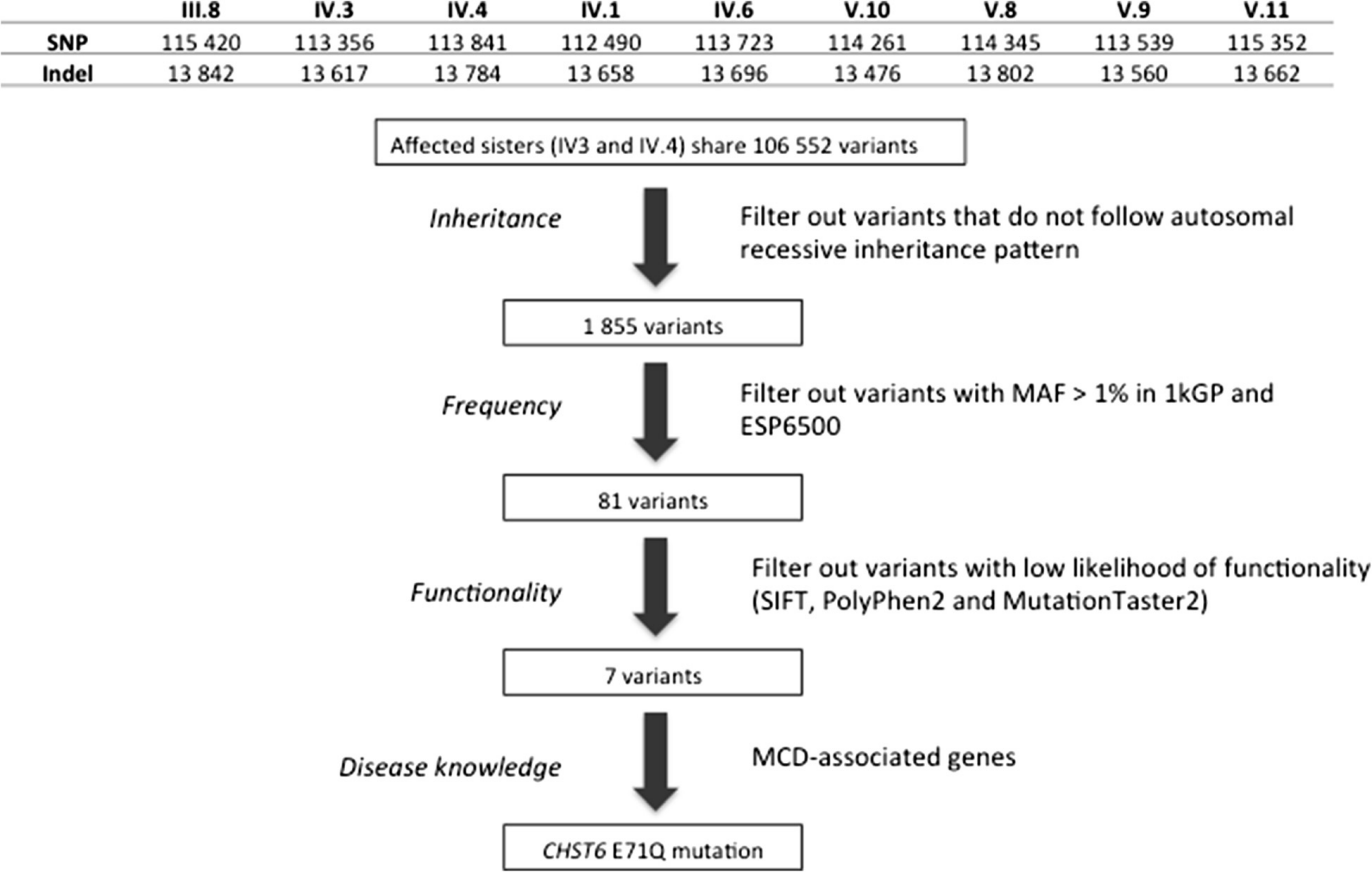
Bioinformatic analysis pipeline

Using a tiered filtering strategy, we first explored variants that followed a simple autosomal recessive inheritance pattern (both affected sisters should be homozygous for the variant allele, carrier mother should be heterozygous and unaffected siblings and children can be either homozygous for the reference allele or heterozygous) (Fig. 2). We only considered novel variants and variants with a minor allele frequency (MAF) of less than 1 % in the 1000 Genomes Project (1kGP) [25] and NHLBI GO Exome Sequencing Project (ESP6500) databases, because developmental eye disorders, and MCD in particular, are considered very rare [26, 27].

Non-coding or synonymous variants were excluded and the variants that remained following the filtering process were evaluated for possible functional impact using SIFT [28], PolyPhen-2 [29] and MutationTaster2 [30]. A database search of the genes containing potential MCD causal mutations was done using PubMed, Online Mendelian Inheritance in Man (OMIM) and the Human Gene Mutation Database (HGMD) Professional 2014.2 [31]. This filtering strategy was then repeated with compound heterozygous mutations that followed an autosomal recessive inheritance pattern in the family.

### Validation and segregation analysis

Sanger sequencing was performed in each of the participating family members to confirm the segregation of the putative MCD-causal variant in the family. A fragment spanning the location of the *CHST6* E71Q mutation was obtained by PCR amplification using sense 5′-AGGTCCAGATCCGTGGGTG-3′ and antisense 5′-CTTTCTGGTTTCCCGGCCA-3′ primers. These primers were then used to sequence the amplicons with the BigDye® Terminator v3.1 Cycle Sequencing chemistry (Thermo Fisher Scientific; Reinach, Switzerland). The purified fragment population was then loaded on a 3730XL Genetic Analyzer for the final capillary electrophoresis step.

### Microscopy

Cross-sections of the corneal buttons excised during penetrating keratoplasty were stained with haematoxylin and eosin (HE). In addition, the following histochemical stains were performed: Alcian blue, Hale’s colloidal iron, Masson’s trichrome, Periodic acid-Schiff (PAS) and Congo red.

## Results

### Clinical evaluation

A South African Sotho-speaking family was identified with two MCD-affected sisters of consanguineous parents at the Eye Clinic of the Charlotte Maxeke Johannesburg Academic Hospital. Both affected sisters (IV.3 and IV.4; Fig. 1) presented to the clinic in September 2007 with the complaint of decreased vision. We recruited seven additional family members as shown in the multigenerational pedigree (Fig. 1).

Patient IV.3 was born in 1977. At the first examination her best-corrected visual acuity in both eyes was counting fingers (<20/200). Both corneas showed ill-defined opacities scattered throughout the stroma. Some were deep and peripheral and typical of macular dystrophy. The corneal stroma between the opacities also showed opacification. Pentacam and contact pachymetry were performed which revealed thin corneas. Corneal astigmatism was present measuring 4.2 dioptres in the right eye and 3.7 dioptres on the left. Intraocular pressures were normal and the eyes otherwise unremarkable, however the fundus view was poor.

Patient IV.4 was born in 1981. The first eye examination was similar to that of her sister, with a best-corrected visual acuity of counting fingers in either eye. Pentacam and contact pachymetry also revealed thin corneas. Corneal astigmatism was 2.4 dioptres on the right and 3.7 dioptres on the left. The intraocular pressures were normal, posterior polar cataracts were present bilaterally and view of the fundi was limited by the cloudy corneas.

Both patients subsequently had uneventful penetrating keratoplasties in their right eyes. The excised corneal tissue was preserved for microscopy and immunohistochemistry. At 3 months post-penetrating keratoplasty patient IV.3 had a clear graft with a best corrected visual acuity of 20/60 in the right eye. We are unsure about age of onset so amblyopia may be a factor in the end result. The lens was clear and fundus examination as well as posterior pole optical coherence tomography (OCT) was normal. Patient IV.4 had a clear graft with a best corrected visual acuity of 20/25 at 2 months post-penetrating keratoplasty in the right eye. Fundus examination and OCT of the posterior pole were unremarkable.

The following unaffected family members all underwent a comprehensive ophthalmological examination and were found to have no ocular pathology:

III.7, III.9, IV.1, IV.6, V.8, V.9, V.10, V.11.

### Microscopy

**Fig. 3:**
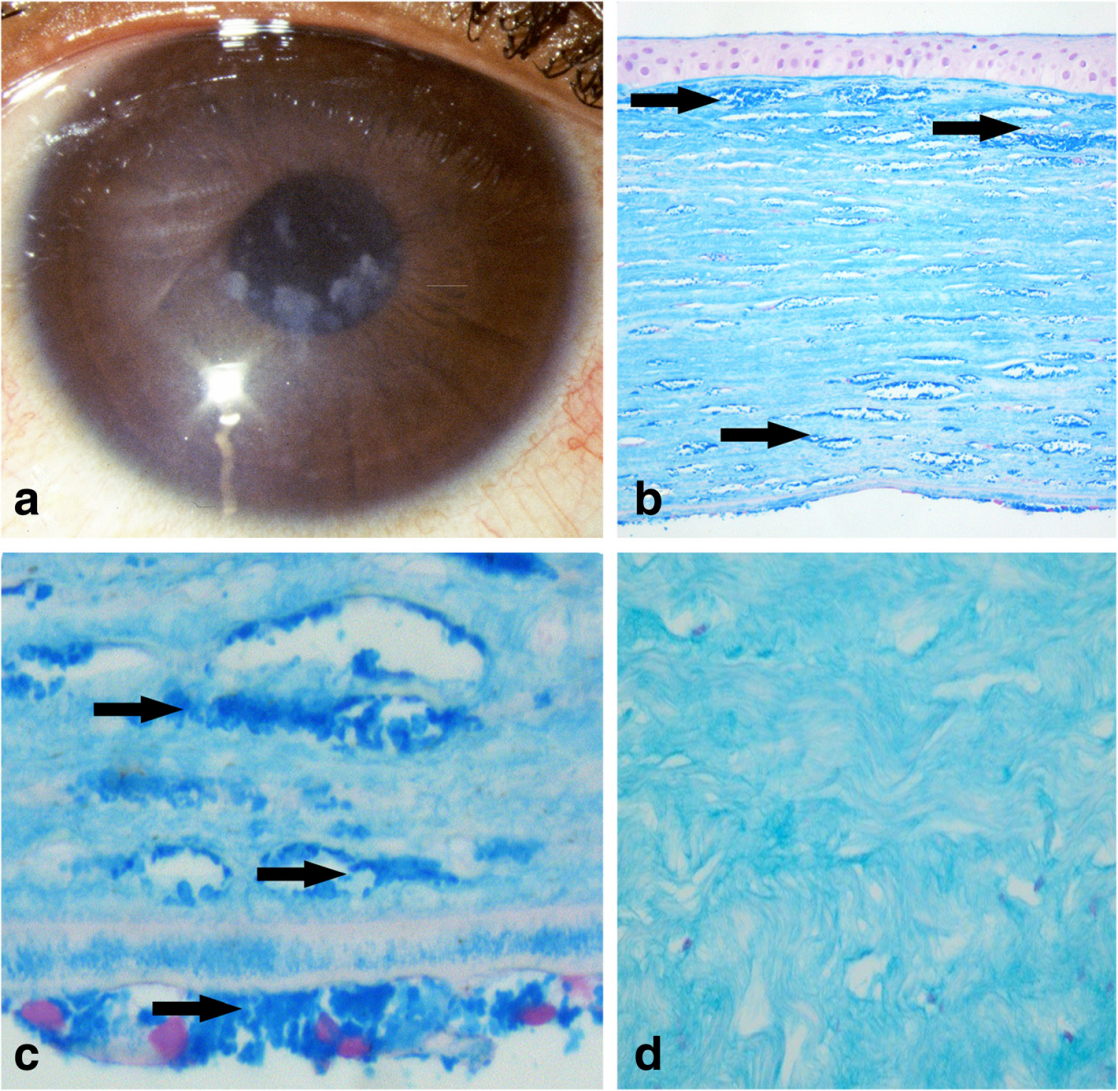
Clinical phenotypes. **a**. Pre-operative photograph of the right eye of IV.3 demonstrating ill-defined corneal stromal opacities. **b**. Cornea of IV.3. Hale’s colloidal iron 20x. The presence of the granular deposits (arrows) are highlighted by a Hale’s colloidal iron stain. **c**. Cornea of IV.3. Hale’s colloidal iron 100x. Higher magnification demonstrates the presence of the granular deposits (arrows) among the lamellae of the substantia propria and within the corneal endothelium. **d**. Control cornea: Hale’s colloidal iron 40x. Control cornea stained with Hale’s colloidal iron fails to identify the presence of basophilic deposits within the substantia propria

Microscopic examination showed tissue compatible with derivation from the cornea (Fig. 3). Granular basophilic deposits were noted among the lamellae of the substantia propria, within the corneal endothelium, Descemet’s membrane and in Bowman’s membrane. The granular eosinophilic deposits were highlighted by Alcian blue, Hale’s colloidal iron and PAS stains. The granules were not seen on the Masson trichrome stain. The Congo red stain was negative. No birefringent material was seen on polarising microscopy.

### Whole exome sequencing and variant filtering

We performed whole exome sequencing on 9 family members to an average depth of 43x. On average, 98 and 95 % of bases were covered to 5x and 10x within the targeted regions, respectively. We identified an average of 127 713 small variants (<50 bp) per individual after filtering out the low quality base calls and variants with a coverage below 5x in the whole family. Approximately 1.2 % of these variants were not present in any of the investigated public databases (Table 1).

**Table 1:** Summary of small variants (<50 bp) identified in the present study

Family member	All variants (% novel)	Missense (% deleterious)	Splice site (% essential)	Frameshift	5'UTR	3'UTR	Stop gained	Stop lost
III.8	129 262 (1.23)	11 250 (27.44)	3 260 (5.58)	202	5 791	32 408	119	58
IV.3	126 973 (1.27)	11 023 (27.59)	3 194 (6.20)	214	5 823	31 995	120	55
IV.4	127 625 (1.27)	11 135 (27.05)	3 149 (5.84)	210	5 733	31 881	114	55
IV.1	126 148 (1.17)	11 006 (27.40)	3 119 (5.67)	206	5 684	31 545	125	60
IV.6	127 419 (1.29)	11 022 (27.05)	3 219 (5.87)	218	5 767	31 843	122	58
V.10	127 737 (1.13)	11 043 (27.45)	3 158 (5.67)	203	5 797	32 413	121	52
V.8	128 147 (1.16)	11 056 (27.30)	3 196 (5.79)	218	5 749	32 288	112	56
V.9	127 099 (1.15)	11 001 (27.52)	3 192 (6.17)	218	5 752	32 165	118	55
V.11	129 014 (1.38)	11 382 (27.56)	3 220 (6.06)	204	5 814	32 512	126	56

The two affected sisters shared 106 552 variants that varied from the human reference genome in either a heterozygous or homozygous state (Fig. 2). This number was reduced to 1 855 variants when we filtered based on a simple autosomal recessive mode of inheritance by only keeping the variants where the affected sisters are homozygous for the variant allele, carrier mother is heterozygous and the unaffected siblings and children are either homozygous for the reference allele or heterozygous. We then retained the 81 variants with a MAF below 1 % in the 1kGP and ESP6500 data sets. Table 2 shows the 7 variants that remained after we filtered out variants with a low likelihood of adverse functionality based on the type of mutation as assessed using SIFT, PolyPhen-2 and MutationTaster2.

**Table 2:** Results form variant filtering aimed at identifying homozygous variants that segregated with MCD in the family with an autosomal recessive inheritance pattern. Only *CHST6* was previously associated with MCD

Gene	Chromosome position^a^	Nucleotide change	dbSNP^b^	Mutation type	SIFT^c^	PolyPhen-2^c^	MutationTaster2
*CSMD1*	8:3351246	G/C	rs368653091	Splice site	--	--	Disease causing (1.00)
*RP1L1*	8:10469917	G/C	rs77585543	Missense	Deleterious (0.00)	Probably damaging (0.99)	Polymorphism (0.99)
*GATA4*	8:11606430	A/G	--	Missense	Tolerated (0.20)	Benign (0.03)	Polymorphism (0.67)
*CHST6*	16:75513516	C/G	--	Missense	Deleterious (0.00)	Probably damaging (1.00)	Disease causing (1.00)
*USP10*	16:84779043	G/A	rs115881577	Missense	Tolerated (0.38)	Benign (0.01)	Polymorphism (0.99)
*CRISPLD2*	16:84900601	A/G	rs114234975	Missense	Tolerated (0.37)	Benign (0.00)	Polymorphism (0.99)
*ANKRD24*	19:4216761	C/T	rs377188730	Missense	Tolerated (0.06)	Benign (0.05)	Polymorphism (0.99)

^a^Chromosomal position given in build 37 format; ^b^Retrieved from bSNP141; ^c^Predictions and scores indicated in brackets retrieved using the Ensembl Variant Effect Predictor

Following the same approach we identified compound heterozygous variants in 9 genes, which followed the autosomal recessive inheritance pattern and where both mutations survived our frequency and functionality filters (Additional file 1: Table S1).

### Putative MCD-causal mutation

**Fig. 4:**
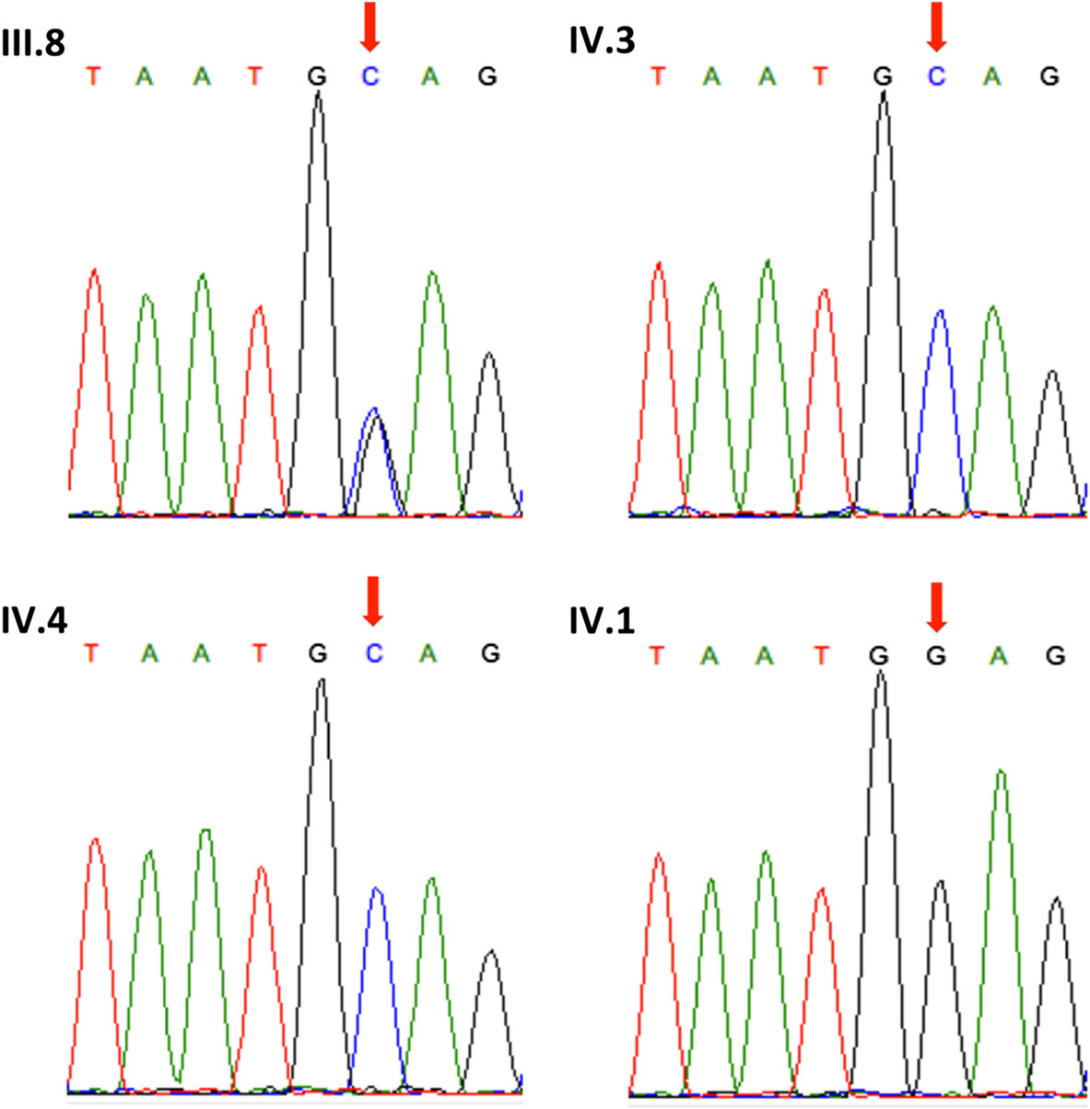
Results form *CHST6* Sanger Sequencing. Electropherograms from Sanger sequencing indicating homozygous E71Q genotype in the two affected sisters (IV.3 and IV.4), heterozygous genotype in the carrier mother (III.8) and homozygous reference genotype in the unaffected brother (IV.1). The red arrow indicates the position of the E71Q mutation and the sequences are given for the reverse strand

The most probable MCD-causal mutation identified is a E71Q (c.211G > C) mutation in the *CHST6* gene, which is a known MCD-causal gene. Sanger sequencing confirmed that this mutation segregated with the disease in the family (Fig. 4) and it was absent from the 1kGP, ESP6500, dbSNP, ClinVar, HGMD and African Genome Variation Project (AGVP) [32] data sets.

This E71Q mutation results in a non-conservative amino acid change as it replaces a negatively charged amino acid (glutamic acid) with an amino acid with polar uncharged side chains (glutamine). All mutations in *CHST6* that alter a codon are presumed to be of pathogenic significance due to the fact that this gene contains large blocks of sequences that are predicted to form a tertiary structural domain that is conserved among sulfotransferase enzymes [1, 5, 18, 33]. The E71 amino acid is conserved in all but two of the human carbohydrate sulfotransferases [5] and falls within the human *CHST6* functional domain (Sulfotransfer_3; ID:PF13469) [34].

### Variants of uncertain significance

We identified potentially damaging homozygous mutations of unknown clinical relevance in *RP1L1* and *CSMD1*, which also segregated with MCD in the family (Table 2). None of the identified compound heterozygous mutations are believed to be of any pathogenic relevance based on gene function, their MAF in the general population and bioinformatic predictors of functional impact (Additional file 1: Table S1).

*RP1L1* is associated with a spectrum of inherited retinal diseases including retinitis pigmentosa and occult macular dystrophy [35, 36]. The S564C mutation identified here is recorded in dbSNP131 (rs77585543) and is predicted to be damaging according to SIFT and PolyPhen-2, but not MutationTaster2. It has a MAF of 4 % in the African sample subset of the 1kGP and two of these individuals are homozygous for this mutation.

We confirmed that *RP1L1* is expressed in retinal cells from healthy donors and found that *RP1L1* expression levels are very low in the cornea, although higher levels of *RP1L1* are detected in the cornea than in negative control samples (human dermal fibroblasts) (Additional file 1: Figure S1). Taken together, these data suggest that the *RP1L1* mutation is unlikely to be the MCD-causal mutation in this family. It is perhaps plausible that this mutation might somehow impact on the retinal phenotype of these patients, but we did not observe any unusual retinal features.

A g.1501249C > G splice site mutation (rs368653091) in *CSMD1* that segregates with MCD in the family is predicted to be disease causing according to MutationTaster2 as it changes the sequence motif adjacent to the acceptor sequence at position 1501247 (Table 2). *CSMD1* encodes the CUB and Sushi multiple domains 1 protein, which is associated with phenotypic variance in neuropsychological disorders [37–39], but it has no documented effect on ocular phenotypes. It is therefore unlikely that this splice site mutation will drive the MCD phenotype in the affected sisters.

## Discussion

We report on a consanguineous black South African family with type I MCD, which is caused by a novel E71Q mutation in *CHST6*. It is the first report of MCD in a Sub-Saharan African family, although it is not the first report of MCD in an individual with African ancestry as Patel et al. previously identified a novel mutation in *CHST6*, which is associated with MCD type II in an African American [40].

The *CHST6* gene encodes the C-GlcNAc6ST enzyme, which is responsible for generating KS in the cornea. Improper functioning of this enzyme leads to malformations in fibril organization in the cornea, which manifests as the characteristic superficial opacities seen in MCD patients [13–15]. However, some studies failed to identify mutations in the *CHST6*/*CHST5* region in MCD patients [1, 5, 6, 9, 18, 19]. We used a whole exome sequencing approach to investigate the cause of MCD in this South African family, because we did not have sufficient African data to inform on whether a candidate gene approach would be successful in this case.

We identified a homozygous E71Q mutation in the two affected sisters that falls within the *CHST6* functional domain, which segregated with MCD in the family. El-Ashry et al. previously identified a G905T transversion that results in another amino acid change at the same position (E71D) in a British family with MCD. However, this mutation was not considered deleterious as it leads to a conservative amino acid change and was inherited in tandem with a P72S non-conservative amino acid substitution and the authors concluded that P72S, rather than the E71D mutation, was the causal mutation in this family [41].

The E71Q mutation identified here results in a change from a negatively charged glutamic acid to the polar uncharged glutamine at a residue that is highly conserved among human sulfotransferases [1, 5]. It is, furthermore, not present in the 1kGP, ESP6500, dbSNP, ClinVar or HGMD databases and three programs for analysing protein functions, Polyphen2, SIFT and MutationTaster2, predicted that the E71Q mutation is probably damaging, deleterious and disease causing, respectively. Taken together, this suggests a very high likelihood that this mutation would have a deleterious effect on the C-GlcNAc6ST enzyme.

We identified two additional variants of unknown clinical relevance that segregated with MCD in our family: a g.1501249C > G splice site mutation in *CSMD1* and a S564C missense muation in *RP1L1*. The *CSMD1* mutation is predicted to be disease causing by MutationTaster2. Aberrant splicing in *CSMD1* has been associated with cancer susceptibility [42], while some studies report association between *CSMD1* mutations and phenotypic variance in neuropsychological disorders [37–39], but it has no documented effect on any ocular phenotype. *In silico* predictions of functionality of splicing variants are, furthermore, more vulnerable to false positive predictions than similar predictors for missense variants [43]. The *CSMD1* splice variant identified here is therefore not the most likely driver of the MCD phenotype in this family.

It is possible to speculate that the *RP1L1* S564C mutation might somehow have an effect on the clinical phenotype in these MCD patients due to *RP1L1*’s association with two retinal disorders, occult macular dystrophy and retinitis pigmentosa. However, we found that this gene is not expressed in the human cornea, making it unlikely to have an impact on the corneal phenotype of this family. We did confirm that this gene is expressed in the human retina, but fundus examination of both individuals post penetrating keratoplasty was normal and the post-operative visual acuities were appropriate for the degree of astigmatism and did not suggest retinal disease. The S564C mutation is also observed twice in a homozygous state in the 1kGP data. Taken together, this suggests that the S564C mutation is unlikely to cause a severe ocular phenotype in this family.

A large number of *in silico* functional predictors are available at present, all of which aim to address the difficult task of prioritizing variants in disease gene identification studies based on different types of data, often yielding different results [44]. However, all of these predictors report an error rate and the studies that aim to evaluate these predictors are slightly flawed due to the overlap in the training and test data sets, which may yield overly optimistic results [45]. It is therefore important to note that *in silico* functional predictors serve as a context within which to interpret sequencing results and that multiple lines of evidence are needed to infer pathogenicity.

African populations contributed the largest number of variants and with the highest fraction of novel variants in the 1kGP [25]. This reflects the great genetic diversity in populations with African ancestry, which has been demonstrated in a number of additional studies [46, 47]. We identified an average of 1569 novel variants per individual. An average of 11 100 of the identified variants were non-synonymous coding (missense) mutations and almost a third of these variants are novel, which is higher than the average recorded in the 1kGP [25].

It is often difficult to get an early, accurate diagnosis for patients with rare diseases, particularly in an overburdened public health care system. The identification of the MCD causal mutation is of great significance to this family as it provides valuable information on the risks to other family members and improves accurate genetic counselling. Fortunately all members of this family that were tested could be reassured that they did not have the same disorder as the two affected sisters. This was especially relevant to the younger family members. Tools for early diagnosis equip the family with the knowledge to seek timely, effective care for other family members with vision problems. Furthermore, our study contributes research into the genetic underpinnings of ocular disease phenotypes in African populations. Studies on African populations have a high probability of identifying novel disease genes due to their high degree of genetic diversity. Such studies additionally serve as a guide to determine where data generated on Caucasian populations might be transferable to Africans.

## Conclusions

We have identified a novel E71Q mutation in *CHST6* as the cause of MCD in this black South African family. This is the first clinical description of MCD in a Sub-Saharan African family as well as the first investigation into the genetic aetiology of MCD in a Sub-Saharan African population. This study therefore contributes towards genetic counselling for MCD patients of African descent.

## Additional file

Additional file 1: Table S1. Results from variant filtering for compound heterozygous that segregated with MCD in the family with an autosomal recessive inheritance pattern. **Figure S1.** Results form qRT-PCR investigating *RP1L1* expression levels in the cornea. (DOCX 121 kb)
