# Supplementary material for: Novel mutation in the CHST6 gene causes macular corneal dystrophy in a black South African family
Source: BMC Med Genet. 2016 Jul 20;17:47. doi: 10.1186/s12881-016-0308-0 (PMC4955246; doi:10.1186/s12881-016-0308-0)
Supplement: Additional file 1: Table S1. — Results from variant filtering for compound heterozygous that segregated with MCD in the family with an autosomal recessive inheritance pattern. Figure S1. Results form qRT-PCR investigating RP1L1 expression levels in the cornea. (DOCX 121 kb) [file 12881_2016_308_MOESM1_ESM.docx]

**Supplementary Table S1** Results from variant filtering for compound heterozygous that segregated with MCD in the family with an autosomal recessive inheritance pattern.

| Gene | Chromosome  position^*^ | Nucleotide  change | dbSNP^†^ | Mutation type | SIFT^‡^ | PolyPhen-2^‡^ | MutationTaster2 |
| --- | --- | --- | --- | --- | --- | --- | --- |
| FAT1 | chr4:187549401 | C/G | rs138797966 | Missense | Tolerated (0.07) | Probably damaging (0.99) | Disease causing (0.99) |
| FAT1 | chr4:187628851 | C/T | -- | Missense | Tolerated (0.21) | Benign (0.01) | Polymorphism (0.99) |
| PPP1R26 | chr9:138378308 | G/A | rs35142799 | Missense | Tolerated (0.68) | Probably damaging (0.99) | Polymorphism (0.99) |
| PPP1R26 | chr9:138379169 | C/T | rs35347705 | Missense | Tolerated (0.42) | Probably damaging (0.96) | Polymorphism (0.99) |
| PRKDC | chr8:48733399 | G/A | rs55644332 | Missense | Tolerated (0.16) | Possibly damaging (0.60) | Polymorphism (0.94) |
| PRKDC | chr8:48739338 | C/A | rs185741285 | Missense | Tolerated (0.54) | Benign (0.01) | Polymorphism (0.99) |
| SH2D3A | chr19:6755137 | G/A | rs143491346 | Missense | Tolerated (0.06) | Possibly damaging (0.47) | Polymorphism (0.99) |
| SH2D3A | chr19:6755221 | G/A | rs148100115 | Missense | Deleterious (0.00) | Probably damaging (0.99) | Disease causing (0.99) |
| SLC25A41 | chr19:6426500 | C/G | -- | Missense | Deleterious (0.02) | Probably damaging (1.00) | Disease causing (0.99) |
| SLC25A41 | chr19:6427433 | C/T | rs78320693 | Missense | Tolerated (1.00) | Benign (0.01) | Polymorphism (0.99) |
| SLC26A8 | chr6:35911783 | A/T | rs145617232 | Missense | Tolerated (0.15) | Benign (0.01) | Polymorphism (0.99) |
| SLC26A8 | chr6:35911871 | C/T | rs113781963 | Missense | Tolerated(0.12) | Benign (0.01) | Polymorphism (0.99) |
| TDRD6 | chr6:46659780 | T/G | rs143054022 | Missense | Tolerated (1.00) | Benign (0.01) | Disease causing (0.76) |
| TDRD6 | chr6:46660495 | C/T | rs151245164 | Missense | Deleterious (0.02) | Probably damaging (0.99) | Disease causing (0.97) |
| UROD | chr1:45479354 | G/A | rs111369324 | Missense | Tolerated (0.59) | Benign(0.01) | Disease causing (0.99) |
| UROD | chr1:45481018 | G/A | rs116233118 | Missense | Tolerated (0.58) | Benign(0.06) | Polymorphism (0.99) |
| ZNRF4 | chr19:5455519 | C/T | -- | Missense | Deleterious (0.03) | Benign (0.02) | Polymorphism (0.99) |
| ZNRF4 | chr19:5455890 | C/G | rs61740899 | Missense | Deleterious (0.04) | Benign (0.06) | Polymorphism (0.99) |

^*^Chromosomal position given in build 37 format; ^†^Retrieved from bSNP141; ^‡^Predictions and scores indicated in brackets retrieved using the Ensembl Variant Effect Predictor

**Supplementary Figure S1.** Results form qRT-PCR investigating *RP1L1* expression levels in the cornea.

Total RNA was isolated from the retina/rpe and cornea tissues from two normal human donor eyes using a Qiagen RNA Mini kit. An equal amount (0.373ug) of RNA from each tissue was used for cDNA synthesis using ABI High-Capacity cDNA Reverse Transcription Kit. TaqMan qPCR was performed with 100ng of cDNA from each tissue using *RP1L1* ABI Gene Expression assay Hs00698865_m1 and beta Actin HS01060665_g1 in triplicate. Average Ct values for each tissue sample were normalized to the average Ct for beta Actin for the same samples to determine the relative amount of *RP1L1*.

Figure S1 indicates that *RP1L1* is expressed in the retina/rpe and corneal tissues from two human donors.
